# Using Tennessee youth hippology contest results as a needs assessment for 4-H horse project members and development of a train-the-trainer program for Tennessee extension agents

**DOI:** 10.1093/tas/txaf068

**Published:** 2025-06-23

**Authors:** Sawyer C Main, Jennie L Z Ivey, Lewrell G Strickland, Justin D Rhinehart, Xiaocun Sun

**Affiliations:** University of Tennessee, Department of Animal Science, Knoxville, TN, USA; University of Tennessee, Department of Animal Science, Knoxville, TN, USA; University of Tennessee, Department of Animal Science, Knoxville, TN, USA; University of Tennessee, Department of Animal Science, Knoxville, TN, USA; University of Tennessee Extension, Office of the Dean, Knoxville, TN, USA; University of Tennessee, Office of Innovative Technologies, Knoxville, TN, USA

**Keywords:** Education, Hippology, Knowledge Assessment, Testing, Youth Contest

## Abstract

Land-Grant Institutions and Cooperative Extension Services seek to disseminate information to the public; however, Extension agents differ in areas of expertise, leaving some counties with minimal ability to provide 4-H horse project members with sufficient content knowledge while agents in other counties are more well versed in equine-specific areas. Results from the 2021 and 2023 Tennessee regional and 2022 Eastern National 4-H Hippology contests were used to determine areas of knowledge deficiency. Nutrition, tack, selection, health, and breeds were categories identified as areas in which 4^th^—12^th^ grade youth lacked adequate knowledge and a training program and new curriculum was developed and delivered to county extension agents in effort to greater their equine knowledge and teaching strategies to disseminate this information to their 4-H Horse Project members and hippology teams. Statistical analysis was conducted using SAS v9.4 (Cary, NC). Nutrition questions were most often missed by senior and junior high youth (k = 7.8, 51.94%; k = 7.5, 44.22%) whereas junior youth missed training questions most frequently (k = 4.9, 54.14%). Of the 5 topic areas of deficiency, selection questions were the lowest percentage missed by senior and junior high youth (k = 5.9, 36.81%; k = 7.1, 39.53%) whereas junior youth missed health questions least frequently (k = 4, 39.87%). It was found that training status had no significant effect on scores from year to year. However, significant effects were found when comparing across question category (*P* < 0.0001), age group (*P* < 0.0001), and year (*P* < 0.0001). Despite the lack of training effect, these findings still prove valuable when assessing performance upholding the extension mission of delivering science-based information to the next generation of industry professionals.

## Teaser

This research identified key knowledge gaps in Tennessee 4-H horse project youth and led to the development of a targeted training program for Extension agents, enhancing equine education across counties. The findings help ensure that youth receive relevant, science-based information to better prepare for future roles in the equine industry.

## INTRODUCTION

### Overview of Tennessee Extension Needs

The University of Tennessee Extension service implements successful youth and adult educational programming to teach and train across a variety of agricultural subject areas. As these programs continue to grow in size and scope, the need arises to train educators on successful delivery and utilization of these programs (Richards et al., 2012). Currently, there is a statewide need for additional species-focused curriculum in Tennessee 4-H programs, based upon County Agent feedback, youth performance at regional, state, and national educational contests, and past animal science-specific in-service trainings.

### Agent Knowledge Deficiency and Overview of Hippology Contests

Extension agents across Tennessee differ in areas of expertise, leaving some counties with minimal ability to provide 4-H members with sufficient content knowledge on equine nutrition, health, and management, while agents in other counties are more well versed in these topic areas. Further, the impact of this knowledge gap on youth enrolled in 4-H programs and the ability to learn, retain, and apply equine management concepts is unknown. Specifically, within the 4-H Horse Project both in Tennessee and across the United States, project members can compete in the 4-H Horse Hippology Contest. This contest is designed to test the knowledge and application skills of horse project members. Typically, participants compete in teams of 4, grouped by age division (Junior, Junior High, and Senior) across several different phases of the contest. The overall goal and scope of these contests are the same. However, the structure and phase compositions differ slightly based on the level of the contest (regional, state, and national). For example, Tennessee regional contests consist of written exam, slides, and judging phases whereas the Tennessee State contest and Eastern National contest are comprised of written exam, slides, judging, and stations phases.

### Methods of Measuring Student Success

Commonly, tests and exams are used to measure student success (Scheerens et al., 2011). Additionally, poor academic performance can be directly related to teaching methods implemented by instructors (Adunola, 2011). However, there has been limited research on using youth educational contests as a means of measuring performance metrics. Determining if educational contests are an appropriate evaluation of student knowledge and skills is pertinent to Extension systems, as minimal literature exists assessing information dissemination, retention, and participant success. Lateral comparison could be made through work evaluating tests and exams as a metric of student success (Scheerens et al., 2011) and a large component of 4-H hippology contest is made up of these evaluation types. This project utilized hippology contests as evaluation tool of student knowledge and took a “Train the Trainer” approach to improving 4-H member’s equine content knowledge of across Tennessee through knowledge empowerment of ANR and 4-H agents.

### Train the Trainer Programs

Extension currently utilizes a train the trainer approach through the annual Joint Animal Science in-service training and monthly hot topics webinars. However, formalized curriculum and assessments regarding end-user knowledge gain, implementation, and application is not as common, and lacking within many livestock project areas. Additionally, the literature has shown that the implementation of Train-the-Trainer programs has yielded positive student knowledge gain (Bess et al., 2003; Orfaly et al., 2005; Pancucci, 2007; Brown et al., 2008; Carruth et al., 2010; Sood et al., 2016). Student preferences differ among learners regarding online and in-person platforms, but both formats can provide successful learning outcomes (Pigg et al., 1980; Neuhauser, 2002; Sajjad, 2010; Adedoyin and Soykan, 2020).

### Study Objectives

The objectives of this study were to assess the effectiveness of using 4-H hippology contests as a means of knowledge assessment for youth and to determine if a train-the-trainer approach is a valuable strategy to equip Extension agents to coach successful hippology teams. We hypothesized that youth hippology contest scores would improve after the implementation of the new training program and curriculum and would improve scores of Tennessee youth when compared to National-level contests.

## MATERIALS AND METHODS

### Regional Hippology Contests

To assess youth knowledge related to equine science and the equine industry within Tennessee, scores were collected from each of the Tennessee Regional 4-H Horse Hippology contests. The University of Tennessee, Knoxville Institutional Review Board determined that the project did not qualify as human subjects research and thus did not require formal approval. The Tennessee Regional 4-H Horse Hippology contest is hosted annually in each of the 3 regions in Tennessee (Eastern, Central, and Western) and consists of 3 phases (University of Tennessee Extension, 2020, 2023). The first phase is a written exam made up of multiple choice, true/false, and matching type questions. Participants in the junior age division are administered a 35-question written exam whereas competitors in the junior-high and senior age divisions receive a 50-question exam. The second phase is a slides phase which requires participants to answer questions based on visual ques shown on a screen. During the slides phase, junior division participants answer 25 questions and those in the junior-high and senior divisions answer 50 questions. All questions for the written exam and slides phases are created by Tennessee Extension staff using approved resources. Resources used for Tennessee are the same as the national contests and are approved by a committee made up of equine extension specialists and faculty members from various states and institutions. In 2021, the Tennessee Regional Hippology contest was administered in a full online format whereas the 2023 regional contest and the 2022 Eastern National contest were delivered in-person. Lastly, all participants compete in a judging phase where they must observe and place 1 halter class and 1 performance class. This information is summarized in Table 1. Specific content and topic areas represented in this contest is selected using the Tennessee 4-H Project Area Outcomes developed in 2017 (University of Tennessee Extension, 2017) (Supplemental File 1).

**Table 1.** Comparison of tennessee regional and eastern national hippology contest

Contest Phase	Tennessee Regional Hippology Contest	Eastern National Hippology Contest
**Age Divisions**	Junior, Junior-High, Senior	Senior only
**Written Exam**	Junior: 35 questions Junior-High: 50 questions Senior: 50 questions	150 questions
**Slide Identification**	Junior: 25 questions Junior-High: 50 questions Senior: 50 questions	50 questions
**Judging**	2 classes (all age divisions)	2 classes
**Stations**	Not included	15 stations (10 questions each)

In 2021 due to residual COVID-19 social distancing restrictions, the Tennessee Regional Hippology contest was administered in a full online format whereas the 2023 regional contest and the 2022 Eastern National contest were delivered in-person. In 2021, the online format was administered using Google Forms, where each region used the same contest format and question order to facilitate the contest per age division. The online format included all written exam and slide identification questions. The 2023 Tennessee Regional Hippology Contest was held in person, where each contestant received identical written exam and slide identification questions for each age division, respectively. Recorded judging classes were used in both the 2023 and 2021 contests and were identical across age divisions. Prior to data analysis, exam phase questions and slides phases questions were categorized into topic areas from the aforementioned project area outcomes.

### Statistical Analysis

**Figure 1.**
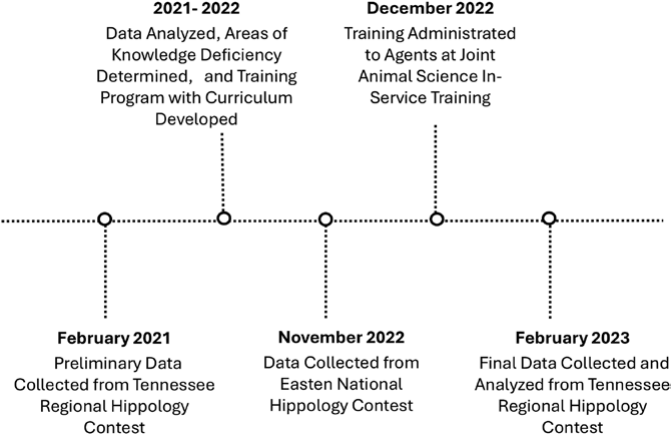
Timeline of data collection and analysis

The timeline of data collection and analysis is represented in Figure 1. Regional contest data from 2021 were analyzed using frequency and means procedures in SAS 9.4 (Cary, NC) to determine a baseline and areas of knowledge deficiency. Analysis determined frequency of incorrect answers for individual questions along with average percentage of incorrect answers for each topic category. Additionally, areas of knowledge deficiency were identified based on this analysis and topic areas were omitted if not similar across all age divisions (Reproduction, Genetics, and Exercise Physiology). All questions were categorized based upon the Tennessee 4-H Horse Project Outcomes and the 5 categories with the lowest performance were considered knowledge deficient (University of Tennessee Extension, 2017). The topic areas represented included colors, behavior, reproduction, ownership, selection (S), breeds (B), facilities, hoof care, tack (T), health (H), nutrition (Nu), waste management, exercise, and genetics.

Youth (n = 224) competed in the 2021 regional 4-H Hippology contest across the Eastern (n = 59), Central (n = 93), and Western (n = 72) regions. Youth competed within their age divisions, where junior (grades 4 and 5, k = 60), junior high (grades 6 to 8, k = 100), and senior (grades 9 to 12, k = 100) divisions competed in written exam and slide identification phases. Scores for each individual contestant were collected and placed into a spreadsheet according to their age division. Columns were labeled with question numbers and the topic area category in which the question was placed. For example, if question #1 pertained to hoof care, it was labeled “Hoof1.” Each row represented a different contestant, and they were given a “1” under each question they answered correctly and a “0” under each question they answered incorrectly. The same process was used to collect scores from the slides phase. Written exam questions and slide questions were considered of equal value and were labeled with the same categories to determine overall performance in each knowledge area.

In January of 2023, data was collected from the 2023 Tennessee Regional Hippology contests where total participants (n = 368) was determined along with overall participant numbers from the Eastern (n = 113), Central (n = 153), and Western (n = 102) regions. Similar to the 2021 regional contests, youth competed within their age divisions, where junior (grades 4 and 5, k = 131), junior high (grades 6 to 8, k = 155), and senior (grades 9 to 12, k = 82) participant numbers were collected. The scores from the 2023 contest were then compared with those from the 2021 contest to determine differences across age division, topic area, and county agent training status. Historical data from the Tennessee State contest has also been included within Table 2.

**Table 2.** Historical data from Tennessee State Hippology contest

Year	Number of Participants
2019	125
2020	135
2021	100
2022	125
2023	137
2024	147
2025	130

### National Contest

The Eastern National Hippology contest is administered in a similar fashion to the regional level contests. However, this contest is only open to competitors in the senior age division who have won at their state level contest and is intended to have increased difficulty. Equine and youth extension specialists from participating states work in conjunction to develop contest material. In the 2022 Eastern National contest, 15 different states were represented. The national level contest still offers the same 3 written phases as the Tennessee Regional contests and administers a written exam phase and slides phase, but the question total is increased to 150 questions on the written exam in multiple choice format only (Eastern National 4-H Horse Roundup, 2024). The slides phase contains 50 questions, matching the regional contests. In addition, participants at the national contest much compete in a stations phase where their knowledge is tested in a more applied technique. Participants rotate through stations and answers questions before the allotted time expires. The stations phase consists of 15 stations with 10 questions at each station resulting in 150 questions answered in this phase (Eastern National 4-H Horse Roundup, 2024). This information is summarized in Table 1. All questions for the Eastern National Hippology contest are generated using the approved resources, in alignment with the Tennessee Regional Hippology contest.

Written exam, slides, and stations phases from the Eastern National contest were categorized according to the Tennessee 4-H Project Area Outcomes like the categorization process implemented for regional contest scores (University of Tennessee Extension, 2017). Additionally, participant answers to each question were give a “1” or “0” for correct or incorrect answers respectively. Total participants in this contest (n = 53) along with contest scores were collected in effort to compare areas of deficiency within Tennessee youth to those across the country.

### Training Content Selection and Format

Once scores were collected and questions labeled with topic area categories, average percentage of correct and incorrect answers for each individual question were calculated in SAS v9.4 using the frequency procedure Upon making this calculation for all questions in the written exam and slides phases for the regional contest and the written exam, slides, and stations phases for the national contest, averages were calculated for questions of the same category. Once this step was completed, the topic areas with the highest percentage of incorrect answers were determined to be areas of knowledge deficiency and became the focus areas for this project. When choosing areas of knowledge deficiency, categories were omitted if they were not present across all age divisions or if they made up less than 5% of the overall composition of the questions (i.e. Genetics was omitted because it is only represented in the senior division and waste was omitted because there were minimal questions asked pertaining to this topic). Overall categories with lacking knowledge included Selection (S), Breeds (B), Tack (T), Health (H), and Nutrition (Nu).

Upon determination of areas of knowledge deficiency, a training program and associated curriculum was created for county Extension Agents to use when coaching their hippology teams for the 2023 contest. Subject matter experts including veterinarians, university faculty members, and extension specialists were selected for each topic and were asked to create educational material associated with that topic to be given to all Extension Agents across Tennessee. Additionally, those subject matter experts developed an in-person training for agents that was implemented at the 2022 Joint Animal Science In-Service training. Agents in attendance not only received the same curriculum pieces but were also taught methods and strategies for implementing the curriculum to hippology teams in their home counties.

### Participant Selection and Determination of Training Status

Annually, the University of Tennessee Department of Animal Science hosts an in-service training for county ANR and 4-H agents where they receive content and training regarding animal science topics across several species including horse, beef, dairy, small ruminant, and poultry. Agents from all 95 Tennessee counties are able but not required to attend. In 2022, 51 ANR and 4-H agents of varying backgrounds and specialty areas were in attendance. Agents attending this training in 2022 were subjected to the train-the-trainer program we developed for this project and received the associated curriculum whereas agents not in attendance only received the curriculum component. Curriculum given to agents included example PowerPoint presentation, ready-made hand-outs and worksheets for students, and introductions to the approved resources used to develop content for hippology contests (Illustrated Dictionary of Equine Terms, Horse Industry Handbook, Feed and Care of the Horse, The Horse 3^rd^ Edition, The Coloring Atlas of Horse Anatomy, Horse Smarts). Trained agents were instructed to utilize delivered content when preparing youth for hippology contests in 2023. The training module administered to agents in attendance delivered content on the specific knowledge gap subjects that were determined from the contest score analysis conducted on the 2021 regional horse hippology contest. Training included presentations with examples and strategy tips for utilizing the curriculum to deliver information to student.

### Final Data Set Analysis

The effects of age division, contest level (state or national), question category, year, and respective interactions between these effects on student performance of answering test questions correctly were analyzed using mixed model logistic regression analysis with test results as the binary outcome variable. Student identifier was modeled as a random block effect. Multiple comparisons were performed with Tukey’s adjustment. Data are presented as counts and percentages. Statistical significance was identified at *P* < 0.05. Analyses were conducted in SAS 9.4 TS1M8 for Windows 64 × (SAS institute Inc., Cary, NC).

## RESULTS

### Summary of Info/Introduction

Preliminary data were collected from the 2021 Tennessee 4-H Regional Hippology contests from each of the 3 regions of the state (Eastern, Central, and Western). For the junior age division, participants were exposed to 10 questions in the health category, 9 questions in the tack category, 11 questions in the nutrition category, 7 questions in the breeds category, and 10 questions in the selection category. For the junior high division, contestants answered 21 questions in the health category, 13 questions in the tack category, 17 questions in the nutrition category, 7 questions in the breeds category, and 18 questions in the selection category. Finally, in the senior division, participants saw 24 questions in the health category, 9 questions in the tack category, 15 questions in the nutrition category, 3 questions in the breeds category, and 16 questions in the selection category. In 2023, the same questions were asked of the age divisions to maintain consistency. Therefore, the quantity of questions answered were the same across each age division (Table 3).

**Table 3.** Number of correct (1) and incorrect (0) responses across all years, categories, and age divisions for Tennessee 4-H Regional Hippology Contests

	Junior				Junior High				Senior			
	2021		2023		2021		2023		2021		2023	
	0	1	0	1	0	1	0	1	0	1	0	1
**Breed**	188	242	461	456	253	356	463	611	87	129	112	134
**Nutrition**	295	384	726	715	654	825	1164	1451	566	524	746	474
**Health**	246	371	740	570	740	1087	1505	1728	831	911	1032	922
**Selection**	285	331	733	577	619	947	1310	1460	427	732	563	742
**Tack**	300	253	720	459	478	653	906	1092	309	340	343	394

### Effects Age Division, Category, Year, and Training Status

Significant effects were found when comparing across question category (*P* < 0.0001) (Table 4), age group (*P* < 0.0001) (Table 5), and year (*P* < 0.0001) (Table 6). Across all age divisions, the chance of students answering breeds questions correctly is 1.162 times as likely as students answering health questions correctly (*P* = 0.0002), 1.202 as likely as answering nutrition questions correctly (*P* < 0.0001), 1.042 times as likely as answering selection questions correctly (*P* = 0.3258), and 1.213 times as likely as answering tack questions correctly (*P* < 0.0001). Additionally, the chance of students answering health questions correctly is 1.035 times as likely as answering nutrition questions correctly (*P* = 0.2539), 1.044 times as likely as answering tack questions correctly (*P* = 0.1935), and 0.896 times as likely as answering selection questions correctly (*P* = 0.0002). Nutrition questions were 0.866 times as likely to be answered correctly when to compared to selection questions (*P* < 0.0001) and 1.009 times as likely to be answered correctly when compared to tack questions (*P* = 0.8005. Additionally, selection questions were 1.164 times as likely to be answered correctly when compared to tack questions (*P* < 0.0001).

**Table 4.** Comparison across question categories of likelihood to have a correct answer

Category 1	Category 2	*P* Value	Odds Ratio
Breed	Health	0.0002	1.162
Breed	Nutrition	< 0.0001	1.202
Breed	Selection	0.3258	1.042
Breed	Tack	< 0.0001	1.213
Health	Nutrition	0.2539	1.035
Health	Selection	0.0002	0.896
Health	Tack	0.1935	1.044
Nutrition	Selection	< 0.0001	0.866
Nutrition	Tack	0.8005	1.009
Selection	Tack	< 0.0001	1.164

**Table 5.** Comparison across age division of likelihood to have a correct answer

Age Group 1	Age Group 2	*P* Value	Odds Ratio
Junior	Junior High	<0.0001	0.575
Junior	Senior	0.1892	0.924
Junior High	Senior	0.0003	1.221

**Table 6.** Comparison of percentage of correct responses from National and State contests

	State (%)	National (%)	*P*-value
**Breeds**	56.93	44.58	0.0242
**Nutrition**	43.20	36.66	0.0583
**Health**	49.59	37.80	< 0.0001
**Selection**	59.82	42.45	< 0.0001
**Tack**	52.96	47.45	0.3762

An effect of year was also observed (*P* < 0.0001) when comparing across all age divisions (Table 6). This effect indicated that that participants were 1.308 times as likely to answer questions correctly in 2021 compared to 2023. Surprisingly, no effect was found when comparing across training status.

When comparing results across age divisions, junior division participants were 0.575 times as like to answer questions correctly of any category when compared to junior high division participants (*P* < 0.0001). When comparing junior division participants to senior division participants, those in the junior age division were 0.924 times as like to answer questions correctly (*P* = 0.1982). Additionally, junior high participants were 1.221 times as likely to answer questions correctly when compared to those in the senior age division (*P* = 0.0002) (Table 5).

### Comparison of Tennessee Regional Scores and Eastern National Scores

In addition to comparing performance across age division, year, and questions category, we also compared the performance of Tennessee senior division participants to those competing at the Eastern National Hippology contest. Overall performance was higher in senior division contestants in the Tennessee contests with 51.39% of answers being correct when compared to 40.47% of answers being correct amongst national contest participants. When comparing performance within each questions category between state and national level contestants, it was observed that state level participants were more likely to answer breeds, health, and selection questions correctly compared to those at the national level. However, there was no significant differences seen in performance across the nutrition and tack categories (Table 6).

## DISCUSSION

Across the state of Tennessee, Extension agents are located in each of the 95 counties to aid in carrying out the Land Grant mission of providing practical knowledge and skills to the common person (Grant et al., 2000; Dailey et al., 2001; Duemer, 2007). However, knowledge, background, and skills of county Extension agents differ vastly, leaving knowledge base gaps among agents and in the information shared to target audiences. Specifically, many counties across the state lack agents with equine-specific knowledge and skills. Therefore, this study utilized the Tennessee Regional Hippology contests as a needs assessment for the Tennessee 4-H Horse program to determine which equine-specific topics reflected the lowest knowledge base amongst youth, and if these areas aligned with areas of deficiency at the Eastern National 4-H Horse Roundup hippology contest. Areas of deficiency were identified within youth contest participants and county agents were given the option to participate in the annual Joint Animal Science In-Service training to learn subject material and teaching strategies to take back to their county and deliver to their hippology teams and horse project groups. The purpose of this training was to implement the effective strategy of providing professional mentorship to educators by providing supplementation related to the identified areas of deficiency and, in turn, improve contest scores in those areas in the year following the training (Sood et al., 2016).

### Differences Between Age Divisions

Overall, junior high division participants (6 to 8 graders) outperformed those of the junior division (4 to 5 graders, Table 5). Likely, junior age division participants have little to no previous exposure to hippology material or subject matter whereas junior-high age division participants may have some previous experience with the contest and the material contained within (Hartley, 1983; Harder, et al., 2005). Therefore, it could be expected for junior division contestants to be outperformed by those of an older age group. Surprisingly, junior-high participants also outperformed senior division participants (9 to 12 graders), which may be influenced by several factors including reduced retention of youth through ages13 to 17 as identified across several states (Table 4, Hartley, 1983; Harder et al, 2005; Newby and Sailee, 2011; Brueler, 2015).

Question difficulty drastically increases between the junior-high and senior division, where more in-depth and applied knowledge is required as divisions progress, in accordance with learning strategies explained in Bloom’s Taxonomy ( Anderson and Krathwohl, 2001, Ivey, et al., 2017). For example, senior division participants are required to know and apply coat color genotypes to real-world examples rather than simply identifying a coat color based on phenotype alone. Results from this study could indicate the need to lower the overall difficulty of senior division questions or provide supplemental instruction at the junior-high and senior divisions to better prepare participants for the new topics and difficulty introduced in the senior division.

To combat the lack of performance from senior division participants, agents and educators may need to implement more effective teaching strategies to ensure that students are fully able to utilize the content. Higher order thinking strategies from Bloom’s Taxonomy like “creating” and “evaluating” should be developed amongst senior division youth (Forehand, 2010). Increases in the presence of advanced concepts in youth curriculum could result in lack of adequate training from some agents. For example, agents may not be familiar with genetic diseases, reproductive physiology, or exercise science and, thus, fail to teach those concepts, or teach at an appropriate depth to their senior hippology teams.

Lack of time and funding, and high work load present barriers for extension agents (Lakai et al., 2012). In some cases, county hippology teams are coached by outside volunteers rather than county agents. County volunteers lack the opportunity to attend the annual Joint Animal Science In-Service Training; therefore, responsibility is placed upon each agent to recruit and train their county volunteers. Knowledge quality, skills, and content may be diluted in this process, especially if County Agents are not well versed in the topic, which may have contributed to the results seen in this study. Further, minimal standards and qualifications exist for county volunteers, resulting in some volunteer-led counties having a volunteer with strong equine knowledge while others may be lacking. Future trainings may benefit from formalized volunteer development content, with a focus on information and resource sharing between counties to combat this inequity (Fulwood and Rowley, 2021).

Hippology contests are purposefully designed to align with youth developmental stages, incorporating increases in content difficulty that reflect age-related differences in cognitive ability, content knowledge, and reading comprehension. This study highlights the importance of age-appropriate assessment strategies and supports the need for differentiated evaluation tools that recognize the developmental continuum from elementary through high school participants.

### Differences Between Question Categories

Across all age divisions, results indicated a higher likelihood of participants answering breed questions correctly compared to all other questions categories (Table 4). Likely, this is due to breed material being relatively easy to understand and memorize and may contribute to lower proficiency in more applied content areas in upper-level age divisions (Ivey, et al., 2017; Hoque, 2018; Güneş, 2020). Further, a national assessment of 4-H Hippology and Livestock Skillathon participants indicated that hippology contest participants gained substantial knowledge in all areas except breed, potentially indicating that breed knowledge was higher at the onset of contest preparation, a phenomenon which may be observed in this study as well (Tomoson and Nold, 2020).

Alternatively, tack related questions reflected the lowest likelihood of being answered correctly by all age divisions (Table 4). The vast array of tack and equipment items covered in approved resources, and thus included in contest materials, differs greatly and spans many disciplines. Some of the more uncommon tack items such as pack saddles or driving equipment may not be the focus of agent instruction due to the lack of commonality in specific geographic areas. Agent discipline familiarity and unintentional agent bias may further explain performance scores in the tack category. Equine savvy agents may compete in or be more familiar with a certain discipline and thus focus more of their instruction toward tack and equipment related to that specific discipline, leading to participants inequitable knowledge of tack and equipment across multiple disciplines. Despite the lack of research pertaining to performance within youth livestock and equine educational contests, these explanations align with previous findings that content contained within the question itself is just as influential as question or stimulus format when evaluating learner performance via written or visual exam (Schuwirth and Van Der Vleuten, 2004).

### Differences Between Year and Training Status

Youth performance within the topic areas of knowledge deficiency were greater in 2021 when compared to 2023 and no effect of training status was observed. Previous research has indicated that train-the-trainer programs prove successful across a variety of industries, including agriculture (Bess et al., 2003; Orfaly et al., 2005; Pancucci, 2007; Carruth et al., 2010; Sood et al., 2016). Additionally, previous research indicates that Extension Agents and agriculture teachers have sufficient knowledge in youth livestock and equine production; however, there is a lack of data-driven studies examining the use of contest scores to assess knowledge retention among youth (Tomoson and Nold, 2020; Strong et al., 2023). Therefore, it was expected that scores would improve between years due to the implementation of agent training and curriculum development but may be explained by several contributing factors.

Contest structure could play a vital role in the overall performance of the youth (Danili and Reid, 2005). In 2021, the Tennessee Regional Hippology contests were held virtually due to the COVID-19 pandemic. During this period, contestants completed the written exam and slide identification phases of the contest through a Google Form. Conversely, 2023 contests were held in-person as they traditionally have been where contestants answer questions from a physical copy of the written exam and view slides in-person.

One limitation of the current study is the confounding of year with contest format. While “year” was initially treated as a time effect in the statistical model, it also inadvertently reflects changes in contest delivery mode—specifically, the 2021 contest was conducted fully online, whereas the 2023 contest was held in person. This introduces a confounding factor, as differences in scores across years may reflect both time and differences in contest format. While the intent of this study was to include the most comprehensive dataset available, this design constraint limits the ability to isolate the effects of contest delivery method independently from year. Nonetheless, the findings offer meaningful insights into participant performance across varying conditions. Future studies should consider a more controlled design that directly accounts for contest format.

Contest structure may have influenced contest results due to participant preferences and learning styles related to online and in-person delivery. In some instances, learners engaging in strictly online education platforms have equal or increased success and satisfaction when compared to face-to-face models (Neuhauser, 2002; Huang, 2020). Conversely, learners in a different study preferred a face-to-face setting, so future research is warranted to determine if and how contest structure impacts contestant performance (Allen et al., 2002; Carrol and Burke, 2010). Participating in a strictly virtual contest could have provided students with comfort or lack of nervousness that is typical with in-person competition and, thus, resulting in higher overall scores in 2023. Despite this limitation, data collection from this study has the potential to provide comparative information on hippology contestant performance in an online setting vs. an in-person model and indicates a baseline knowledge value for the impact of training effectiveness within Tennessee.

Additionally, the set of youth that participated in the 2021 contests and the 2023 contests are likely composed of different individuals due to participants aging out, new youth entering the contest, and teams being comprised of different youth sets. However, collecting data from different sets of youth provides a more all-encompassing representation of population dynamics, retention, and overall performance within the hippology contests that occur naturally throughout age divisions and within county and state programs (Harder et al., 2005; Newby and Sallee, 2011).

A “lag effect” from the training provided during this study may also have impacted results. The agent training and introduction of supplemental curriculum was provided in December 2022, prior to the regional contests that took place in February of 2023. It is likely that coaches may not have been able to adequately provide instruction on these target content areas prior to the 2023 contest. Additionally, agent trainings are only held once per year which may not coincide with hippology contest meetings in each county and thus limit the overall effectiveness of the training when comparing scores from year to year. The literature suggests that training shows greater overall increased performance when evaluated using lagged time periods which could explain the lack of training effect found when evaluating scores within the first year of our study (Chen, et al., 2013). However, the training still provided agents with valuable curriculum and strategies to implement in the future. Future evaluation of contest scores could yield a training effect due to the additional time granted for agents to implement the aforementioned materials and teaching strategies. Additionally, future data collection over a span of multiple years could prove helpful in making more meaningful conclusions based on training impact and repeatability.

Finally, agent turnover could also play a role in affecting the overall scores in the observed areas of deficiency. Within the state of Tennessee and the surrounding states, it is common for counties to see changes in agent employees frequently (Mowbray, 2002; Ezell, 2003). Furthermore, this high turnover rate could have left some counties without equine-focused programs and create new horse projects in other counties between 2021 and 2023, creating inconsistencies within the knowledge base of Tennessee youth. The 2023 UT Extension Impact Report shares that 32 new agents were hired across Tennessee from 2021-2022, which may have influenced the impact of the training delivered and subsequently assessed at the Regional Hipplology contests in January 2023 (UT Extension, 2023). Additionally, newer agents may be unfamiliar with the process of accessing horse project and hippology resources through SharePoint and could cause their hippology teams to lack adequate training and knowledge. Despite the high agent turnover rate and common unfamiliarity with hippology resources, this project succeeded in providing participating counties with valuable knowledge, skills, and resources to implement and reference within their hippology teams and 4-H horse project groups.

### Difference in Senior Scores Between State and National

An overall difference in performance was observed when comparing senior competitors from the Tennessee state contests to those in the Eastern National contest (Table 6). It is important to note that the Tennessee Regional contests and the Eastern National contest shared the same approved reference material for content generation (Illustrated Dictionary of Equine Terms, Horse Industry Handbook, Feeding and Care of the Horse (2^nd^ Edition), The Horse (3^rd^ Edition), The Coloring Atlas of Horse Anatomy, and Horse Smarts: An Equine Reference & Youth Activity Guide). It was shown that state level participants had higher performance in all question categories (Table 6). Most likely, this is due to the significant increase in content difficulty between the Tennessee regional contests and the Eastern National contests. Further, contest preparation for National level contests may provide a substantial increase in youth knowledge comparatively to regional state contests, and can indicate the need for states to provide resources to agents, volunteers, and coaches earlier to aid in knowledge gain and retention (Tomoson and Nold, 2020).

Additionally, the structure of the Eastern National and Tennessee Regional Hippology contests is slightly different. At the Eastern National contest, participants must rotate through stations and answer questions independently without the ability to converse and seek help from their teammates, thus adding increased stress, nervousness, autonomy. Comparatively, the Tennessee Regional contests do not contain stations and team problem phases, and the written exam phase contains less questions than the Eastern National. Although the contest phases differ, data used within this study addressed topic area performance, rather than phase performance, for consistency in collection, assessment, and comparison between each contest.

### Implications and Conclusions

Despite the widespread popularity implementation of equine educational events such as hippology contests, there remains a substantial lack of peer-reviewed literature evaluating their educational outcomes, the training of Extension agents and volunteers, and the overall impact on youth knowledge gain. This study contributes to filling that gap by offering a foundational analysis of how these programs function across varying regions and age divisions. The complexity of interpreting 4-H program data is compounded by the decentralized nature of delivery and variation in program structure, which can lead to differences in participant experience and achievement. Our work begins to dissect these complexities, offering an easier understanding of how youth equine programs are implemented and evaluated.

Findings from this study hold value when assessing the current state of the Tennessee 4-H Horse Project and could prove to be valuable across other youth contests and state horse projects if applied. The methods used in this study can provide insight into knowledge gaps within youth curriculum and program delivery regardless of the subject matter or content area. Additionally, if implemented yearly, this model could prove useful in providing long term tracking of performance data for any youth organization, contest, or project group both within and outside of an extension setting. Our findings yielded that participant scores did not significantly increase after the implementation of the train-the-trainer program. However, this study has strong practical merit, particularly in the context of real-world administration of hippology contests. Given the variability in contestant experience, contest administration, and volunteer involvement across counties and regions, there is a clear need for consistent, data-driven approaches to assess educational outcomes. This work illustrates how contest data, which is already collected regularly and across age divisions, can serve as a consistent and reliable tool for identifying trends in youth learning and agent development. By utilizing these existing data sources, Extension professionals can make informed decisions about agent and volunteer training, curriculum updates, and contest organization, while also building a foundation for program evaluation and continuous improvement.

In addition to its evaluative findings, this study offers a framework for how Extension programs can strategically use existing contest data as a form of educational assessment. Rather than requiring the creation of new, standalone evaluation tools, Extension professionals can utilize data generated from contests such as hippology to gauge learning progression, identify educational gaps, and guide future program improvements. This approach provides a method for integrating program evaluation into ongoing educational efforts, enhancing the sustainability of youth development initiatives within Extension.

## Supplementary Material

txaf068_suppl_Supplementary_Materials

## CRediT Roles

S. Main; conceptualization, methodology, investigation, writing original draft, writing review and editing, visualization

J.L.Z. Ivey; conceptualization, methodology, investigation, writing review and editing, supervision, project administration

L. Strickland; conceptualization, methodology, investigation, writing review and editing

J. Rhinehart; conceptualization, writing review and editing

X. Sun; methodology, formal analysis, software, writing review and editing
