# Supplementary material for: Using Tennessee youth hippology contest results as a needs assessment for 4-H horse project members and development of a train-the-trainer program for Tennessee extension agents
Source: Transl Anim Sci. 2025 Jun 23;9:txaf068. doi: 10.1093/tas/txaf068 (PMC12207865; doi:10.1093/tas/txaf068)
Supplement: txaf068_suppl_Supplementary_Materials [file txaf068_suppl_supplementary_materials.pdf]

W 466-A

1-2 YEARS IN PROJECT

Horse

November 2017
For more information, contact:
Jennie Ivey, Department of Animal Science
Jennifer Richards, Tennessee 4-H Youth Development
James W. Swart, Tennessee 4-H Youth Development

Educational Topics

General knowledge

- Define colt, filly, foal, weanling, yearling, mare, gelding, stallion.
- Understand how horses are measured for height.

Breeds and registries

- Identify three to five pony, light, sport and draft breeds (12-20 total) within the US and their respective registries.

Colors

- Identify basic coat colors and facial and leg markings.

History of the horse

- Understand general evolutionary changes of equids through time.
- Identify differing working and recreational roles of horses through time.

Selection and use of horses

- Identify common parts of horse's body encompassing the head, neck, fore and hind legs, abdomen, and hindquarter.
- Understand general concepts of conformation and balance.
- Recognize two to three common conformation faults.

Hoof care

- Identify major parts of the hoof.
- Determine why regular hoof care is important to the health of the horse.

Equine health and disease

- Assess horse's temperature, respiratory rate, hydration status and heart rate.
- Identify symptoms of a sick, injured or unwell equid that would require medical attention.
- List three to five diseases or conditions that affect horses.
- List two to three vaccines horses receive annually (core or risk-based).

Tack and equipment

- Identify parts of an English (hunt seat, cutback and dressage) saddle and Western saddle, and appropriate bridles.
- Understand difference between curb and snaffle bits and identify one to two examples of each.

Nutrition and feeding

- Understand differences between forage and concentrates and their role within an equine diet; provide two examples of each.
- Identify physical qualities of hay indicating poor or high quality.
- List basic rules and guidelines of feeding horses (i.e., amount of minimum vs. recommended forage intake per day).

Responsible horse ownership

- Understand the needs of the horse from an owner.
- Identify basic, minimum care standards for horses and other equids.

Horse behavior

- List horse's senses and associated structures.
- Understand horse's ability to process information and apply to maneuvering around horses safely.
- Discuss horse as a prey animal and role of herd in innate behavior.

Educational Contests

Horse judging

- Youth should be able to accurately place halter and performance classes in accordance with the Tennessee 4-H Horse Championships Handbook in practice and at county, regional, open state contests.
- Youth should be preparing and delivering simple sets of oral reasons to accurately reflect placing decisions in practice and at county, regional and open state contests.

Hippology

- Youth should be comfortable completing a 35-question written exam, 25-slide identification phase, and judging two classes of performance and/or halter classes in accordance with the Tennessee 4-H Horse Championship Handbook.
- Youth should be studying above outcomes in preparation for contests at the county, regional and state levels.

Horse Bowl

- Youth should be studying above outcomes in preparation for a quiz game show-style contest in accordance with the Tennessee 4-H Horse Championships Handbook for contests at the county, regional and state levels.

Public speaking

- Youth should practice preparing and delivering speeches to county agents, volunteers and/or other adult or youth leaders on content outcomes listed above in accordance with the Tennessee 4-H Horse Championships Handbook.

Individual presentation

- Youth should practice preparing and delivering individual presentations to county agents, volunteers and/or other adult or youth leaders on content outcomes listed above in accordance with the Tennessee 4-H Horse Championships Handbook.

Team presentation

- Youth should practice preparing and delivering team presentations to county agents, volunteers and/or other adult or youth leaders on content outcomes listed above in accordance with the Tennessee 4-H Horse Championships Handbook.

For youth participating in riding activities or showing, the following concepts should be mastered in accordance with years in project, but are not considered mandatory.

AG.TENNESSEE.EDU
Real. Life. Solutions.™

W 466-A 11/17 18-0151 Programs in agriculture and natural resources, 4-H youth development, family and consumer sciences, and resource development. University of Tennessee Institute of Agriculture, U.S. Department of Agriculture and county governments cooperating. UT Extension provides equal opportunities in programs and employment.

Horse

November 2017
For more information, contact:
Jennie Ivey, Department of Animal Science
Jennifer Richards, Tennessee 4-H Youth Development
James W. Swart, Tennessee 4-H Youth Development

Educational Topics

General knowledge

- Broaden vocabulary, descriptive terms and knowledge of the equine industry though mastery of the outcomes detailed below.

Breeds and registries

- Identify 5-10 pony, light, sport and draft nationally and internationally (20-40 total) along with their respective registries.
- Determine use of breed and their application to the equine industry.
- Understand differences between light breed types, such as hunter-, stock- and saddle-types, and provide examples of each.

Colors

- Analyze and identify coat color modifications and coat patterns.

History of the horse

- Delineate the scientific classification of the horse and other equids.

Selection and use of horses

- Select horse based on conformation, form to function and defend reasoning.
- Evaluate horse's conformation and performance through judging comparison.
- Understand differences between conformation faults and blemishes; provide a variety of examples for each.
- Identify parts of body systems, muscles, bones and ligaments through internal and external structure assessment.
- Recognize common fore and hind limb conformation faults that can cause unsoundness or lameness.

Hoof care

- Determine main diseases and conditions that affect hoof health and quality, along with potential therapeutic interventions.
- Identify main parts of a horse shoe and nail.
- Name three to five tools used by a farrier and their uses.

Equine health and disease

- Categorize and describe major equine diseases based on vector, transmittance, symptoms and treatment
- Categorize and describe major internal and external parasites affecting equids.
- Identify difference between core- and risk-based vaccinations for horses.
- Know basic equine first aid and emergency care.
- Relate condition of horse's teeth to overall health.
- List signs of colic and laminitis, methods of treatment, and management strategies for prevention or care of each condition.
- Understand preventative health care practices for horses and develop a management plan including vaccinations, fecal egg counts/deworming, dental care and other associated procedures.

Tack and equipment

- Discuss basic equipment needed for trailering and safety during transportation.
- Understand importance of tack and equipment safety and identify situations where tack could be considered dangerous.
- Identify types of materials bits are made from and factors influencing bit severity.
- Recognize different types of saddles, head stalls, supportive equipment, rider turnout and other basic equipment relative to specific disciplines.

Nutrition and feeding

- Describe characteristics of acceptable and unacceptable forages, concentrates and water for equine consumption.
- Identify sources of protein, fat, fiber and carbohydrates from commonly utilized feedstuffs from feed tag/ingredient list and feed samples.
- List three poisonous/toxic plants and describe their characteristics.
- Identify fat and water-soluble vitamins.
- Categorize minerals as macro- or trace minerals and identify which minerals belong to which classification.
- Understand basic concept of maintenance nutritional needs over nutritional needs of growth, pregnancy, lactation and work.
- Apply and utilize concepts of body condition scoring and body weight estimates to make simple ration recommendations.
- Recognize two to three weeds and two to three forage species commonly seen in horse pasture.

Responsible horse ownership

- Determine cost of horse ownership on annual basis and create accurate estimates of ownership and care.
- Apply concepts of horse welfare to management concerns throughout equine industry, including the unwanted horse population.
- Identify criteria for selecting horses for purchase or lease based on experience, age and suitability of mount to rider.
- Identify criteria for selling horses and reasonable estimation of training and cost.

Horse behavior

- Classify roles of horses within a herd, and relate to pecking order for management considerations.
- Understand how horse communicates through position of eyes, ears, head/neck, hind quarter, tail, and overall body position

Facilities

- List three types of fencing used for horse pastures and provide advantages and disadvantages for each.
- Identify types of horse bedding, including pros, cons and disposal of each type.
- Understand emergency preparedness and disaster planning.
- Discuss factors impacting pasture quality, overgrazing and soil erosion.

Waste management

- Assess waste produced by horses annually and methods of disposal.

Educational Contests

Horse judging

- Youth should be able to accurately place halter and performance classes in accordance with the Tennessee 4-H Horse Championships Handbook in practice and at county, regional and open state contests.
- Youth should be preparing and delivering simple sets oral reasons to accurately reflect placing decisions in practice and at county, regional and open state contests.

Hippology

- Youth should be comfortable completing a 50 question written exam, 50 slide identification phase, and judging two classes of performance and/or halter classes in accordance with the Tennessee 4-H Horse Championship Handbook
- Youth should be studying above outcomes in preparation for contests at the county, regional and state levels.

Horse Bowl

- Youth should be studying above outcomes in preparation for a quiz game show-style contest in accordance with the Tennessee 4-H Horse Championships Handbook for contests at the county, regional and state levels.

Public speaking

- Youth should practice preparing and delivering speeches to county agents, volunteers and/or other adult or youth leaders on content outcomes listed above in accordance with the Tennessee 4-H Horse Championships Handbook.

Individual presentation

- Youth should practice preparing and delivering individual presentations to county agents, volunteers and/or other adult or youth leaders on content outcomes listed above in accordance with the Tennessee 4-H Horse Championships Handbook.

Team presentation

- Youth should practice preparing and delivering team presentations to county agents, volunteers and/or other adult or youth leaders on content outcomes listed above in accordance with the Tennessee 4-H Horse Championships Handbook.

For youth participating in riding activities or showing, the following concepts should be mastered in accordance with years in project, but are not considered mandatory.

AG.TENNESSEE.EDU

Real. Life. Solutions.™

W 466-B 11/17 18-0151 Programs in agriculture and natural resources, 4-H youth development, family and consumer sciences, and resource development. University of Tennessee Institute of Agriculture, U.S. Department of Agriculture and county governments cooperating. UT Extension provides equal opportunities in programs and employment.

Horse

November 2017
For more information, contact:
Jennie Ivey, Department of Animal Science
Jennifer Richards, Tennessee 4-H Youth Development
James W. Swart, Tennessee 4-H Youth Development

Educational Topics

General knowledge

- Broaden vocabulary, descriptive terms and knowledge of the equine industry though mastery of the outcomes detailed below.

Breeds and registries

- Understand basic foundation breeds, origin, and history of breeds in the US and their role within the equine industry
- Identify 10-15 pony, light, sport and draft breeds nationally and internationally and their respective registries.
- Apply breeds to various disciplines and their suitability for work/performance.

Colors

- Determine inheritance of coat colors through genetic coding of coat colors.
- Relate coat colors to breed standards.
- Understand use of markings for identification purposes, including tattooing and branding.

History of the horse

- Identify and describe the evolutionary forms of *Equus*.
- Understand the role of subspecies that contributed to ancestry of today's horse.

Selection and use of horses

- Create sound, accurate and detailed reasons for evaluation of horse conformation and performance classes.
- Apply concepts of form to function, use of horses and breed characteristics to assess suitability of stock for breeding, performance or culling, and adherence to breed standards.
- Understand how conformation faults and blemishes can impact a horse's gait, soundness and overall health.
- Identify internal and external parts of the horse's anatomy and relevant applications to physiology including but not limited to muscles, bones, tendons/ligaments, teeth, body systems, and associated structures.

Hoof care

- Identify various types of horse shoes and their applications in therapy, performance and maintenance.
- Understand the application of identifiable farrier tools along with their uses in hoof management and care.
- Recognize hoof diseases, conditions and structural issues and propose potential solutions.

Equine health and disease

- Describe life cycles, symptoms and prevention strategies including management practices for internal and external parasites in equids.
- Explain differences in metabolic, non-infectious and infectious diseases and conditions in horses including modes of transmission, symptoms, treatment, containment, prevention and other associated details where appropriate.
- Create management plans including biosecurity measures and disease control for horse facilities.
- Understand and apply first aid and emergency management techniques to various settings and situations, including identification of first aid kit components and their uses.
- Apply knowledge of dental conditions (i.e., parrot or monkey mouth, wave mouth, hooks/points) to horse's health and management.

Tack and equipment

- Recognize and apply tack and equipment to respective disciplines and uses, including but not limited to boots, bits, saddles, bridles, rider turnout, artificial aids and training tools.

Nutrition and feeding

- Create nutritional feeding management plans based on body weight, body condition, age and disease, with consideration given to maintenance and/or increased nutritional needs (i.e., growth, breeding, lactation, work load, or disease).
- Understand and apply differences in concentrate and fiber feedstuffs based on their suitability within an equine diet (i.e., high vs. low protein, fat, fiber, simple or complex carbohydrate) and other characteristics of feedstuffs.
- Identify toxic plants to horses, describe their effects if horses are exposed, and physical descriptions of each.
- Distinguish common weeds, grasses and legumes based on their growth periods, physical appearance, management strategies and nutritive value through growth stages.
- Understand the role of fat- and water-soluble vitamins within the body and function of each vitamin within horse's physiology.
- Describe role of macro and trace minerals within the body and impact on physiological function.

Responsible horse ownership

- Determine role of horse processing on unwanted horse population.
- Understand responsible breeding and impact on equine population in US and internationally.
- Select horse(s) based on criteria relating to horse age, training and suitability for rider's age, experience level, and goals; assess cost and market for sale based on similar parameters

Horse Behavior

- Apply herd dynamics to feral and domestic horse herds and role within management practices.

Facilities

- Discuss safety practices to be implemented when designing or building equine facilities and structures, including but not limited to fire prevention, building materials, dimensions, ventilation.
- Identify characteristics that are advantageous/disadvantageous for site selection of equine facilities.
- Discuss barn and arena design and materials, including flooring; stall layout and size; walls; roofing; ventilation; storage for bedding, hay and equipment; human facilities; and other structures associated with equine facilities.
- Review and apply pasture management considerations to farm management plans and recommendations for maintaining or establishing new pasture.
- Create a budget for facility construction and maintenance based on material estimates.
- Understand paperwork, permits and other regulations impacting equine facility construction.
- Analyze the impact of horse farms on environmental quality and ways to improve management practices.

Waste Management

- Develop plans for waste management including composting of manure, urine, stall waste and carcasses.
- Understand limitations, benefits and drawbacks of composting, burial, incineration, biodigestion and other methods of waste disposal.

Exercise Physiology

- Understand light, moderate, heavy and very heavy workload classifications with regard to workload descriptions, discipline examples, heart rates and nutritional needs.
- Understand differences within skeletal muscle fiber type, metabolism and function.
- Develop training programs for specific disciplines, with consideration given to specificity and activity level.

Genetics

- Understand and apply central dogma of genetic information and modes of inheritance to equine examples.
- Determine role of genetics on inherited diseases including dominant vs. recessive linkage, symptoms of disease, breeds affected, and responsible breeding techniques to avoid transmittance.

Reproduction

- Recognize structures of mare and stallion reproductive anatomy.
- Identify signs of parturition, stages of parturition, and indicators of situations where veterinary intervention is warranted (i.e., red bag, dystocia).
- Identify, define and describe role of reproductive hormones and endocrinology in creating gametes, regulating reproductive cycles and onset of sexual maturity.
- Describe how sperm and ova are formed, along with the stages of fertilization, implantation and maternal recognition of pregnancy.
- Understand components of a breeding soundness exam and impact of sperm motility and morphology on fertility.
- Recognize birth defects, nutritional deficiencies and other conditions affecting foals.
- Understand reproductive management concepts including estrus detection, mare and stallion management, and other related topics.
- Describe reproductive technologies utilized within the equine industry including live cover, artificial insemination, embryo transfer, cloning and semen collection.

Educational Contests

Horse Judging

- Youth should be able to accurately place halter and performance classes in accordance with the Tennessee 4-H Horse Championships Handbook in practice and at county, regional and open state contests.

- Youth should be preparing and delivering simple sets oral reasons to accurately reflect placing decisions in practice and at county, regional and open state contests.

Hippology

- Youth should be comfortable completing a 50-question written exam, 50-slide identification phase, and judging two classes of performance and/or halter classes, six stations, along with a prepared and impromptu team problems in accordance with the Tennessee 4-H Horse Championship Handbook.
- Youth should be studying above outcomes in preparation for contests at the county, regional and state levels.

Horse Bowl

- Youth should be studying above outcomes in preparation for a quiz game show-style contest in accordance with the Tennessee 4-H Horse Championships Handbook for contests at the county, regional and state levels.

Public Speaking

- Youth should practice preparing and delivering speeches to county agents, volunteers and/or other adult or youth leaders on content outcomes listed above in accordance with the Tennessee 4-H Horse Championships Handbook.

Individual Presentation

- Youth should practice preparing and delivering individual presentations to county agents, volunteers and/or other adult or youth leaders on content outcomes listed above in accordance with the Tennessee 4-H Horse Championships Handbook.

Team Presentation

- Youth should practice preparing and delivering team presentations to county agents, volunteers and/or other adult or youth leaders on content outcomes listed above in accordance with the Tennessee 4-H Horse Championships Handbook

- For youth participating in riding activities or showing, the following concepts should be mastered in accordance with years in project, but are not considered mandatory.

AG.TENNESSEE.EDU

Real. Life. Solutions.™

W 466-C 11/17 18-0151 Programs in agriculture and natural resources, 4-H youth development, family and consumer sciences, and resource development. University of Tennessee Institute of Agriculture, U.S. Department of Agriculture and county governments cooperating. UT Extension provides equal opportunities in programs and employment.
